# Phytohormone Interaction Modulating Fruit Responses to Photooxidative and Heat Stress on Apple (*Malus domestica* Borkh.)

**DOI:** 10.3389/fpls.2017.02129

**Published:** 2017-12-14

**Authors:** Carolina A. Torres, Gloria Sepúlveda, Besma Kahlaoui

**Affiliations:** ^1^Facultad de Ciencias Agrarias, Universidad de Talca, Talca, Chile; ^2^Centro de Pomaceas, Facultad de Ciencias Agrarias, Universidad de Talca, Talca, Chile

**Keywords:** sunburn, sunscald, ACC, abiotic stress, ABA, jasmonic acid, auxin, ethylene

## Abstract

Sun-related physiological disorders such as sun damage on apples (*Malus domestica* Borkh) are caused by cumulative photooxidative and heat stress during their growing season triggering morphological, physiological, and biochemical changes in fruit tissues not only while it is on the tree but also after it has been harvested. The objective of the work was to establish the interaction of auxin (indole-3-acetic acid; IAA), abscisic acid (ABA), jasmonic acid (JA), salicylic acid (SA), and ethylene (ET) and its precursor ACC (free and conjugated, MACC) during development of sun-injury-related disorders pre- and post-harvest on apples. Peel tissue was extracted from fruit growing under different sun exposures (Non-exposed, NE; Exposed, EX) and with sun injury symptoms (Moderate, Mod). Sampling was carried out every 15 days from 75 days after full bloom (DAFB) until 120 days post-harvest in cold storage (1°C, > 90%RH). Concentrations of IAA, ABA, JA, SA, were determined using UHPLC mass spectrometry, and ET and ACC (free and conjugated MACC) using gas chromatography. IAA was found not to be related directly to sun injury development, but it decreased 60% in sun exposed tissue, and during fruit development. ABA, JA, SA, and ethylene concentrations were significantly higher (*P* ≤ 0.05) in Mod tissue, but their concentration, except for ethylene, were not affected by sun exposure. ACC and MACC concentrations increased until 105 DAFB in all sun exposure categories. During post-harvest, ethylene climacteric peak was delayed on EX compared to Mod. ABA and SA concentrations remained stable throughout storage in both tissue. JA dramatically increased post-harvest in both EX and Mod tissue, and orchards, confirming its role in low temperature tolerance. The results suggest that ABA, JA, and SA together with ethylene are modulating some of the abiotic stress defense responses on sun-exposed fruit during photooxidative and heat stress on the tree.

## Introduction

Plants are sensitive to environmental stresses such as chilling, high light, supra-optimal temperatures, drought and salinity, among others. These conditions are major limiting factors for growth and geographic distribution (Boyer, 1982). To face various environmental stresses, plants adapt through a range of physiological and biochemical changes. Although the response of plants to abiotic stress depends on various factors, plant hormones are considered the most important endogenous substances that modulate physiological and molecular responses, key for plant survival as sessile organisms (Wolters and Jurgens, 2009; Vob et al., 2014; Wani et al., 2016).

In order to survive under changing environments, plants need to regulate their growth and development (Wolters and Jurgens, 2009). Phytohormones, a diverse group of signaling molecules found in small quantities in cells, mediate this regulation. Their pivotal roles in promoting plant acclimation through modifying growth, development, source/sink transitions, and allocation of nutrients have been well established (Fahad et al., 2015). Phytohormones act at their site of synthesis or elsewhere in the plant after being transported (Peleg et al., 2011; Wani et al., 2016). Moreover, they are of key importance in plant development and phenotypic plasticity. They include auxin (IAA), abscisic acid (ABA), ethylene (ET), salicylic acid (SA), and jasmonates (JAs). Some phytohormones, such as ABA, have been identified as stress hormones. ABA plays critical roles in plant development: maintenance of seed dormancy, inhibition of germination, growth regulation, stomatal closure, fruit abscission, besides mediating abiotic and biotic stress responses (Yang et al., 2011). Kim et al. (2016) reported that JA has a major role in fungal infection, as well as modulating other abiotic stress (low and high temperature, ozone, and wounding) response mechanisms, and growth and development processes such as flowering, tuber formation, and regulation of tendrils (Kim et al., 2016).

According to Brodersen et al. (2005), SA is a stress hormone found in many plant species with a key role in the hypersensitive response (HR) or systemic acquired resistance (SAR). In addition to this defense mechanism, SA can modulate other physiological responses such as thermogenesis, ion absorption, and programmed cell death. As other phytohormone, ethylene (ET), as mediator in the senescence process, is also related to diverse abiotic and biotic stresses responses. This phytohormone is involved in different phases of plant growth as leaf and petal abscission, fruit ripening, and flower senescence.

Auxin is one of the most multi-functional phytohormone and is vital not only for plant growth and development but also for governing and/or coordinating plant growth under stress conditions (Kazan, 2013; Wani et al., 2016). Though there has been a recent upsurge in our understanding of auxin regulation of plant growth and development, its role as a regulator of stress response is still little understood. However, auxin stimulates the transcription of a large number of genes called primary auxin response genes, and these genes have been identified and characterized in several plant species including rice, Arabidopsis, and soybean (Javid et al., 2011). Auxin is regarded as an influential constituent of defense responses via regulation of numerous genes and mediation of crosstalk between abiotic and biotic stress responses (Fahad et al., 2015; Wani et al., 2016). However, identification of novel genes involved in stress responses may prove to be a vital target for engineering abiotic stress tolerance in important crops.

Apple (*Malus domestica* Borkh.) is a major fruit crop grown in Chile. The production area has a semi-arid climate with high solar irradiance and elevated temperature during the growing season. This climate lead unavoidably to abiotic stress in fruit trees causing biochemical and physiological changes in fruit tissues resulting in visible and invisible sun damage symptoms caused by photooxidative and heat stress (Torres et al., 2006), some of which are tissue discoloration and browning of the fruit surface directly exposed to sunrays, changes in fruit shape and texture, decrease of water content, and increase is sugar concentration (Torres et al., 2013).

Upon sun exposure, up-regulation of antioxidants systems, such as the ascorbate-glutathione cycle (Ma and Cheng, 2003; Torres et al., 2006; Chen et al., 2008), phenylpropanoids and carotenoids synthesis and accumulation (Ma and Cheng, 2003; Torres et al., 2006; Chen et al., 2008; Felicetti and Schrader, 2008; Yuri et al., 2010; Tartachnyk et al., 2012), and accumulation of compatible osmolytes occurs (Torres et al., 2013). These and others defense mechanisms during high light and heat stress on fruit are most certain regulated by phytohormones, transducing stress signals throughout tissues, although limited information is available (on apple fruit). Torres et al. (2013) found significantly higher ‘stress’ ethylene early in the season during sun damage development. Whether this occurs by ET crosstalk with other phytohormones known to modulate abiotic stress responses in plants is still unknown.

The aim of this work was to understand how stress phytohormones interact during photooxidative stress leading to sun-related disorders pre- and post-harvest on apples in order to build strategies to enhances crop resistance.

## Materials and Methods

### Plant Material and Fruit Sampling

Apples (*Malus domestica* Borkh.) cv. Granny Smith from two commercial orchards located in San Clemente, Maule valley, Chile (‘Quilpue,’ 35°30′26,92′′ S., 71°25′25,07′′ W, 229 m.a.s.l, and ‘Los Lirios’, 35°31′18,13′′ S., 71°26′32,25′′ W, 230 m.a.s.l) were sampled during 2013/2014 season. Fruit with different sun exposures to direct sunlight and photooxidative injury levels were sampled every 15 days from 75 to 165 days after full bloom (DAFB), and after commercial harvest (165 DAFB) during cold storage at 1°C (>90% RH) for 120 days. At each time point, 4 replicates of 5 fruits each per sun injury category (= treatments) were used for statistical analysis.

Fruit classification was done according the following categories: ‘Non-exposed’ (NE), tissue non-exposed to direct sunlight (shaded); ‘Exposed’ (EX), tissue exposed to direct sunlight without visual symptoms of sun-damage; ‘Moderate’ (Mod), discolored tissue showing yellowing and browning classified as moderate sun-injury symptoms.

Within 24 h of sampling fruit, peel (4–5 layers of epidermal and hypodermal cells, <1 mm thick) of 5 fruit per category (composite sample) was removed with a scalpel, immediately flash frozen with liquid N2, and stored at -80°C until analysis.

### Auxin, Jasmonic Acid, Salicylic Acid, and Abscisic Acid Determinations

Tissue extraction was carried by a modified method from Gómez-Cadenas et al. (2002) and Durgbanshi et al. (2005) Frozen tissue (0.5 g FW-1) was extracted with 80% methanol (5 mL) using mortar and pestle, after centrifugation (5.000 g × 10 min) supernatant was dried using N_2_ gas. After re-suspension using 2 mL water:pure diethyl-ether (1:1) samples are shaken for 1 min. The diethyl-ether fraction was subsequently dried using N_2_ gas and re-suspended in 10% methanol (200 μL). A 10 μL volume was injected to a UHPLC-mass spectrometer (Orbitrap, Thermo Scientific, Waltham, MA, United States). After 20 min run, quantification was carried out using pure standards of indole-3-acetic acid, (±)-abscisic acid (ABA), (±)-jasmonic acid, and salicylic acid (Sigma–Aldrich, St. Louis, MO, United States) using Xcalibur v.2.13 (Thermo Scientific, Waltham, MA, United States).

### Internal Ethylene Concentration (IEC)

Internal ethylene concentration (IEC) was performed according to Hernandez et al. (2014). One ml of gas from the core of the fruit was taken through the calix end and then injected into a Hewlett Packard, Series II HP 5890 gas chromatograph equipped with a packed column [Porapak Q (80°C)] and a flame ionization detector with Helium as a carrier gas. Ethylene concentration was determined using a proper standard curve.

### ACC and Malonyl-ACC (MACC) Concentrations

ACC and MACC were determined according to Bulens et al. (2011). Around 0.25 g FW-1 frozen ground peel was extracted with 5% (v/v) sulfosalicylic acid (SSA). After vortexing the mixture, it was shaken at 4°C for 30 min. The extract was then centrifuged at 3.090 × *g* at 4°C. One and half mL of supernatant was used to determine free ACC. 20 mM HgCl2 (0.2 mL) were added to the aqueous sample in a glass vial. After sealing it, a mixture of NaOH-NaOCl (2:1, v/v) (0.1 mL) is injected. After 4 min and vortexing, a 1 mL ethylene sample was taken from the vial and injected into a Hewlett Packard, Series II HP 5890 gas chromatograph equipped with a packed column [Porapak Q (80°C)] and a flame ionization detector.

For total ACC another 1.5 mL of supernatant of the extract was subjected to acidic hydrolysis using 6M HCl (0.2 ml) for 3 h in a water bath at 99°C. The sample was then centrifuged at 22.000 × *g* for 5 min and the supernatant collected. 0.1 mL of supernatant diluted with 0.6 mL of distilled water was mixed with 20 μL ACC standard solution (50 μM), as well as 10 mM HgCl2 (0.1 mL) to proceed with the sealing of the glass vial. A mixture of NaOH-NaOCl (2:1, v/v) (0.1 mL) is injected to the vial. After 4 min and vortexing, a 1 mL ethylene sample was taken from the vial and injected into a Hewlett Packard, Series II HP 5890 gas chromatograph equipped with a packed column [Porapak Q (80°C)] and a flame ionization detector. MACC is calculated subtracting Total ACC with free ACC.

### Experiment Layout and Statistical Analysis

Data analyses was carried out using one-way analysis of variance (ANOVA), after normality assumptions were met (Levene’s test). Sun exposure and sun injury categories at each time point were considered treatments. Mean differences were separated using Tukey’s multiple range test (HSD, *P* ≤ 0.05). GLM procedures using SAS statistical software (version 8.02; SAS Institute, Cary, NC, United States) were used.

## Results

### Characterization of Phytohormones during Fruit Development

**FIGURE 1:**
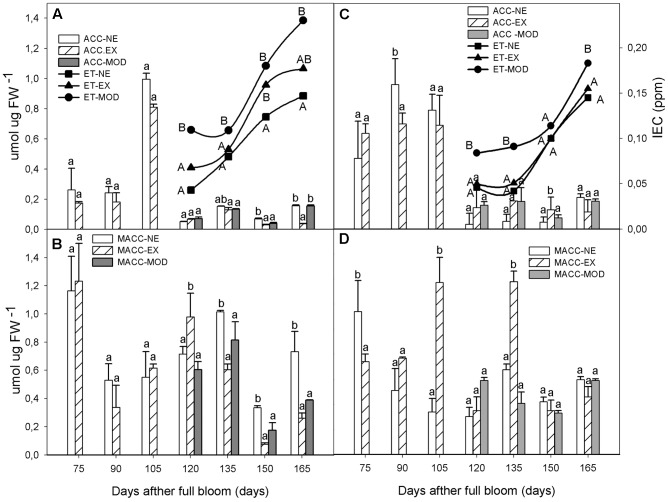
Internal ethylene concentration (ET), free ACC and Malonyl-ACC (MACC) interactions in apple skin during fruit development of non-exposed (NE), exposed (EX), and moderate sun-damaged (MOD) fruit from orchards Quilpue **(A,B)** and Los Lirios **(C,D)**, Maule Region, Chile. Different upper-case (ET) and lower-case (ACC and MACC) letters indicate statistical difference (*P* ≤ 0.05) between sun damage levels at each time point. Values represent means ± SE of 3 biological replicates.

After 120 DAFB, when Mod symptoms developed, ET concentration increased (26% in average in both sites) compared to NE and EX levels (**Figures 1A,C**), although not statistically significant. As expected, in all categories ET levels increased exponentially as fruit matured (**Figures 1A,C**).

Regarding ACC levels, prior to Mod symptoms appearance, tissue showed higher ACC concentrations regardless of sun exposure (105 DAFB; **Figures 1A,C**). After this time point (at 120 DAFB), ACC levels decreased more than 90% until 165 DAFB in all sun exposure categories.

MACC were higher than ACC levels throughout fruit development varying between sun exposure categories (**Figures 1B,D**). In Quilpue site, the highest were observed early in the season at 75 DAFB, decreasing thereafter (**Figure 1B**). Non-sun injured tissue (NE, EX) had, in general, higher MACC levels than Mod category (**Figures 1B,D**). After 120 DAFB, MACC remained significantly higher than ACC until 165 DAFB, regardless of sun exposure (**Figure 1**).

**FIGURE 2:**
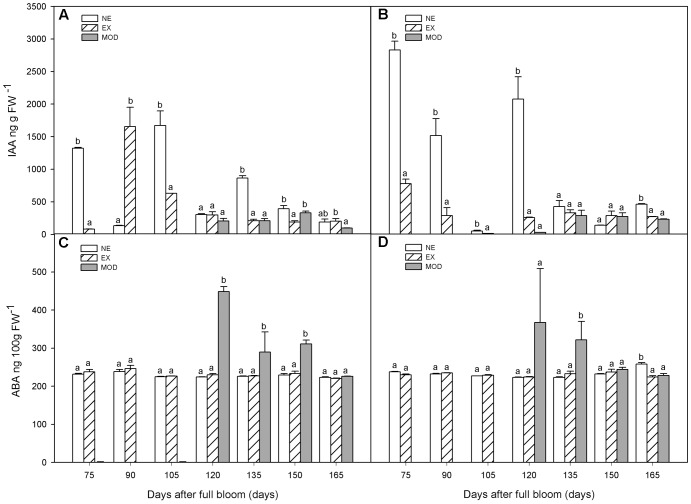
Indol acetic acid (IAA) **(A,B)** and abscisic acid (ABA) **(C,D)** concentrations in apple skin during fruit development of non-exposed (NE), exposed (EX), and moderate sun-damaged (MOD) fruit from orchards Quilpue **(A,C)** and Los Lirios **(B,D)**, Maule Region, Chile. Different lower-case letters indicate statistical difference (*P* ≤ 0.05) between sun damage levels at each time point. Values represent means ± SE of 3 biological replicates.

Although unexposed tissue (NE) tended to have higher levels of IAA early in the season compared to EX, it was only statistically significant at 75, 105 DAFB, and after 135 DAFB (**Figures 2A,B**). Sun-injured tissue (Mod) had, in general, lower IAA levels compared to NE, but similar to those of EX’s (**Figures 2A,B**). On NE and EX, IAA decreased as the season progressed, which was not observed in Mod category (**Figures 2A,B**).

There was a significant increase (around twofold) in ABA levels when sun injury appeared (Mod) after 120 DAFB, although not always statistically different between categories (**Figures 2C,D**). In NE and EX, ABA concentrations remained stable throughout fruit development (**Figures 2C,D**). At 120 DAFB, ABA as well as ET where significantly higher in Mod category compared to NE and EX (**Figures 2C,D**).

**FIGURE 3:**
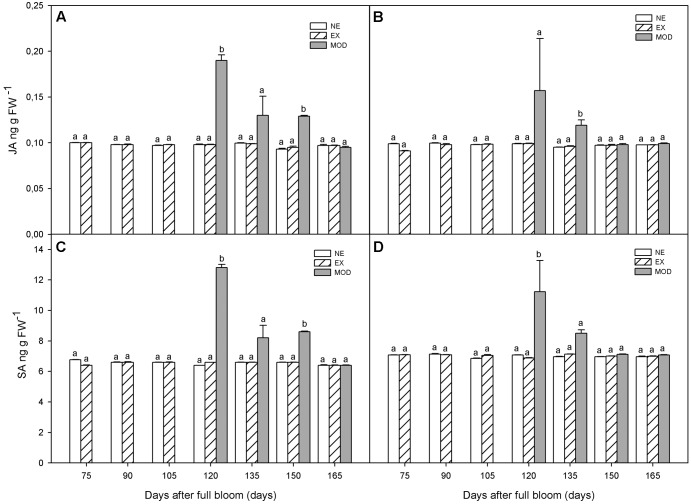
Jamonic acid (JA) **(A,B)** and salicylic acid (SA) **(C,D)** concentrations in apple skin during fruit development of non-exposed (NE), exposed (EX), and moderate sun-damaged (MOD) fruit from orchards Quilpue **(A,C)** and Los Lirios **(B,D)**, Maule Region, Chile. Different lower-case letters indicate statistical difference (*P* ≤ 0.05) between sun damage levels at each time point. Values represent means ± SE of 3 biological replicates.

Jasmonic acid remained at a low level throughout fruit development (**Figures 3A,B**). As well as ABA, SA, and ET, JA was significantly higher (75% in average) in Mod tissue, in both experimental sites, compared to NE and EX, and decreased (44% in average) toward harvest (**Figures 3A,B**). SA showed similar behavior as to ABA during fruit development (**Figures 3C,D**). When sun injury appeared (Mod) at 120 DAFB, SA was significantly higher in this tissue compared to NE and EX (**Figures 3C,D**).

### Characterization of Phytohormones Post-harvest

**FIGURE 4:**
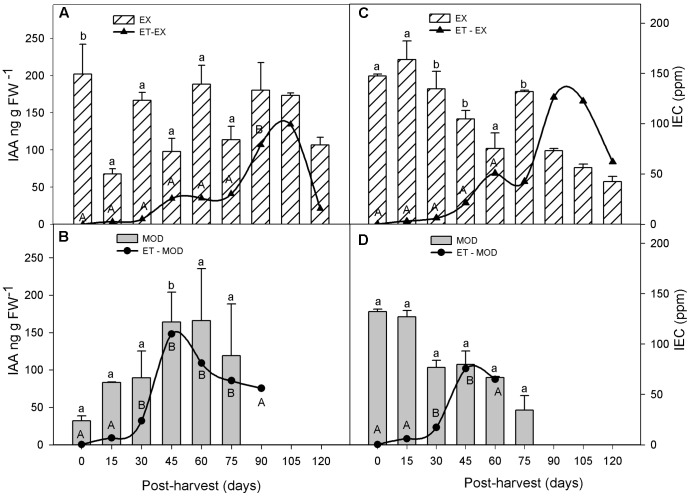
Indol acetic acid (IAA) and ethylene (ET) evolution in apple skin during low temperature storage (0°C, >90%RH) of exposed (EX; **A,C**) and moderate sun-damaged (MOD; **B,D**) fruit from orchards Quilpue **(A,B)** and Los Lirios **(C,D)**, Maule Region, Chile. Different upper-case and lower-case letters indicate statistical difference (*P* ≤ 0.05) between sun damage levels (**A** vs. **B** and **C** vs. **D**) at each time point. Values represent means ± SE of 3 biological replicates. MOD fruit was available until 75 days into cold storage.

During cold storage after fruit harvest, ET in Mod fruit remained significantly higher than that in EX but only until 75 days into storage (**Figure 4**). ET peak in Mod fruit occurred as early as 4 weeks prior to that of EX fruit (**Figure 4**).

In general, IAA concentrations remained low post-harvest (<200 ng gFW-1) (**Figure 4**). Although IAA levels were overall 20% lower in sun injured fruit (Mod) compared to undamaged ones, these were statistically similar, except between 30 and 45 days in ‘Los Lirios’ site (**Figures 4C,D**).

**FIGURE 5:**
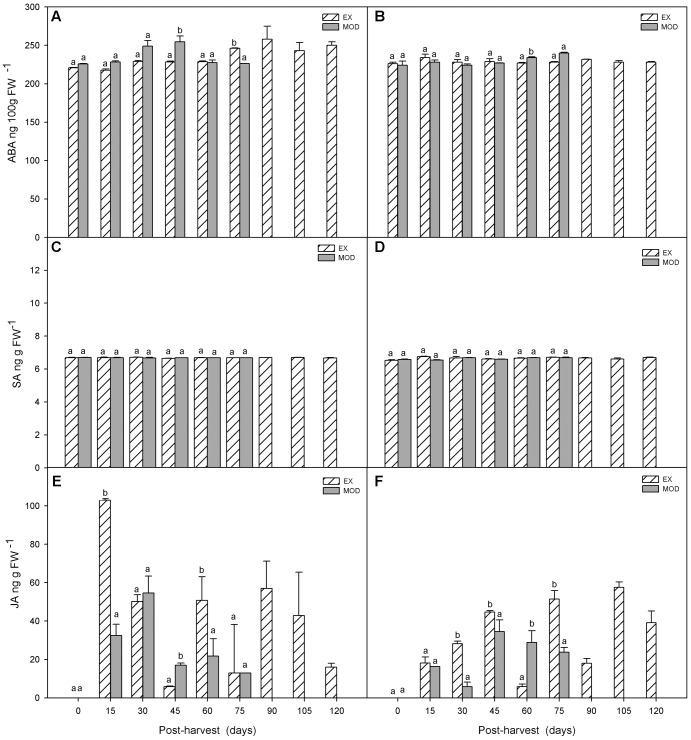
ABA **(A,B)**, SA **(C,D)** and JA **(E,F)** concentrations in apple skin during low temperature storage (0°C, >90%RH) of exposed (EX) and moderate sun-damaged (MOD) fruit from orchards Quilpue **(A,C,E)** and Los Lirios **(B,D,F)**, Maule Region, Chile. Different lower-case letters indicate statistical difference (*P* ≤ 0.05) between sun damage levels at each time point. Values represent means ± SE of 3 biological replicates. MOD fruit was available until 75 days into cold storage.

Similar to what occurred pre-harvest, ABA levels did not change during cold storage in EX fruit, but differences between EX and Mod tissue, as well as between sites, disappeared post-harvest (**Figures 5A,B**). SA levels remained stabled throughout cold storage in EX and Mod (**Figures 5C,D**).

Finally, in Quilpue site, JA increased around 12-fold and 7-fold in EX and Mod, respectively, within 15 and 30 days in EX and Mod, respectively, after fruit was placed in cold storage (**Figure 5E**). In Los Lirios site, JA level did not have a clear pattern although gradually increased in the first 45 days into cold storage (**Figure 5F**).

## Discussion

Sun-related physiological disorders such as sun damage on apples (*Malus domestica* Borkh), are caused by cumulative photooxidative and heat stress during the growing season, causing morphological, physiological, and biochemical changes in fruit tissues (Racsko and Schrader, 2012). Furthermore, this physiological disorder alters fruit quality after cold storage, particularly due to browning of the skin, once low temperature stress has been imposed (Hernandez et al., 2014; Torres et al., 2016).

Phytohormones play a key role modulating different resistance systems against such dangerous conditions (Peleg and Blumwald, 2011; Verma et al., 2016; Wani et al., 2016). In Torres et al. (2013) it is suggested that photooxidative stress response in apple fruit may be, in part, regulated by ethylene. In their study, sun exposed tissue also showed lower relative water content (<9–10%) and more negative tissue water potentials (<28%) compared to undamaged one, as well as osmolytes synthesis and accumulation (i.e., carbohydrates), indication of dehydration stress and osmoregulation. In fact, dehydration in grapes after harvest has shown to induce genes involved in ET synthesis (ACO), carbohydrates, and polyphenols metabolisms (Rizzini et al., 2009). Furthermore, Bonghi et al. (2012) found that phenylpropanoids were differentially accumulated during post-harvest grape dehydration, i.e., accumulation of flavonols and *trans*-resveratrol and decreased of flavan-3-ols, procyanidin B1 and B2. This phenomenon is also a well-known stress defense mechanism against sun exposure on apples (Racsko and Schrader, 2012), and as well as in grapes, specific flavonols are accumulated, such as quercetin glycosides (Yuri et al., 2010).

In agreement with Torres et al. (2013), we also found significantly higher levels of ET early in the season on sun injured fruit (Mod) compare to non-injured ones (**Figure 1**), which remained for the rest of the growing season and after harvest during cold storage (**Figure 4**). Moreover, Mod fruit exhibited also an earlier post climacteric decreased of ET, which was around 4 weeks prior to that observed in undamaged fruit (EX) (**Figure 4**).

Although ET is known for its important role on plant growth and development, and ripening and senescence in climacteric fruit (Yang, 1995; Saltveit, 1999; Paul et al., 2012), sun injured fruit does not ripen earlier than non-injured one. In fact, Schrader et al. (2009) and Torres et al. (2013) found higher flesh firmness in sun injured fruit than in undamaged ones in various apples cultivars. In our study, we found that this phenomenon carries over post-harvest (data not shown), indicating that perhaps sun damage triggers changes in cell wall components that alter tissue softening, as it does on pears (Raffo et al., 2011), or accumulates lignin components that increases tissue firmness (data not shown). In either case, ET could be the signaling molecule. This topic needs to be further studied.

An increase in ACC prior to ET was also observed in sun injured tissue (**Figure 1**). In rosemary (*Rosmarinus officinalis*) ACC levels have been positively correlated with solar irradiation, but not to drought (Munné-Bosch et al., 2002).

Auxins have a widely known role regulating plant growth and development (Ostrowski et al., 2016). Nonetheless, only recently studies have also highlighted their role in stress response (Shi et al., 2014; Ostrowski et al., 2016). Auxins in association to ethylene regulate root development and architecture, a key event in drought (Shi et al., 2014) and salinity tolerance (Ostrowski et al., 2016). Auxin has also been found to inhibit ET synthesis (Leyser, 2010; Wang and Irving, 2011). In our research, IAA level was the lowest in sun injured tissue (Mod, **Figures 2A,B**), which was the one that had the highest ET concentration (**Figure 1**). These results indicate that IAA and ET are perhaps crosstalking in a signaling network regulating defense responses against photooxidative and heat stress on apple fruit.

In fact, ET and auxin crosstalk in Arabidopsis have been positively correlated with flavonol biosynthesis and accumulation (quercetin and kaempferol) (Lewis et al., 2011), a known photoprotective mechanism in sun exposed apple fruit (Racsko and Schrader, 2012), as well as in other fruit crops (Mahouachi et al., 2014). In particular, the accumulation of quercetin glycosides have been directly correlated with sun damage severity (Felicetti and Schrader, 2010; Yuri et al., 2010; Hernandez et al., 2014; Torres et al., 2016).

In Arabidopsis, both phytohormones induced flavonol accumulation via independent signaling pathways by increasing the abundance of transcripts of different enzymes and transcriptional regulators in their biosynthetic pathway, such as *MYB12* (common for both hormones), *PAP1* and *TTG1*. These flavonoids, and in particular quercetin and its derivatives, were found to modulate root growth and auxin basipetal transport (Lewis et al., 2011). Recently, in Arabidopsis shoots was reported that kaempferol 3-*O*-rhamnoside-7-*O*-rhamnoside acts as an endogenous inhibitor of auxin transport (Yin et al., 2014). Moreover, light-induced quercetins and kaempferols accumulation in root cells of Arabidopsis causes asymmetrical cell elongation through their accumulation at the side closer to light exposure (Silva-Navas et al., 2016).

The increase or decrease in auxin levels under certain abiotic stresses it seems to be organ and specie specific. *Lupinus albus* leaves showed low IAA levels after water stress, although the opposite was found in roots (Pinheiro et al., 2011). Mahouachi et al. (2014) found high IAA levels after drought periods on banana plants. In our work, although dehydration stress is present during sun damage development on exposed fruit tissue (Torres et al., 2013), IAA levels where the lowest in sun injured tissue (Mod, **Figures 2A,B**).

High ET levels on sun injured fruit early in the season (120-135 DAFB) were positively correlated with high ABA concentrations (**Figures 2C,D**). This could indicate that ABA may be triggering ET synthesis as a stress-response mechanism. In fact, exogenous ABA treatments have shown to increase ET production in many species (Riov et al., 1990; Gómez-Cadenas et al., 1996; Sharp et al., 2000; Jiang and Joyce, 2003) including apples (Lara and Vendrell, 2000). This relation was not observed in undamaged tissue (NE, EX), which is in agreement with Lara and Vendrell (2000) results on Granny Smith flesh. These authors also found an increase in endogenous ABA together with ACC, MACC, and ET levels, but only in the flesh.

Abscisic acid is known to have an important role in water stress signaling pathways in plants (Zhu, 2002; Wilkinson and Davies, 2010; Mahouachi et al., 2014). This phytohormone not only accumulate under drought conditions, but it appears to have an important role in post-stress recovery of the tissue (Munné-Bosch et al., 2002).

In citrus fruit, ABA deficiency resulted in altered water relations in the flavedo, increased water loss, reduced peel firmness and enhanced rot incidence, especially at 12°C. Under high rate of water loss, ethylene production significantly increased in ABA-mutant fruit, demonstrating that ABA would negatively regulate water stress-induced ethylene in citrus fruit (Alferez et al., 2005). As indicated by Romero et al. (2013), downstream of ABA signaling, post-harvest water deficit would activate phospholipase activity and trigger lipid catabolism in plasma membranes of citrus fruit. Thus, ABA may be a key regulator of post-harvest water-deficit response mechanisms in this species.

Besides the known role of SA in seed germination, photosynthesis, transpiration, stomatal closure, thermogenesis, cell growth, ion uptake and local and systemic resistance to diseases (Clarke et al., 2004), this phytohormone has been found to have an important role enhancing resistance to various biotic and abiotic stresses, as well as in anti-ripening and anti-senescence mechanisms, all of them important topics for post-harvest management in horticultural crops (Asghari and Aghdam, 2010). Our results showed a burst in SA at 120 DAFB along with ethylene in sun injured tissue (**Figures 3C,D**). This may indicate that SA is acting, along with ET and ABA, in a local tolerance network against photooxidative and heat stress. In fact, according to Asghari and Aghdam (2010) and Asghari and Hasanlooe (2015), there is an initial increase in SA concentration during local and systemic acquired resistance to disease and stress in resistant crops. Similar results have been reported in banana leaves in response to water stress (Mahouachi et al., 2014). SA levels remained stable throughout cold storage (**Figures 5C,D**) indicating that photo-oxidative and heat stress on fruit while in the tree would be responsible for SA transient increase, but not low temperature stress imposed post-harvest, although free SA has been implicated in cold stress tolerance regulation in *Hordeum vulgare*, wheat and grape (Kosová et al., 2012; Mutlu et al., 2013). External applications of methyl salicylate on kiwifruit (*Actinidia deliciosa*) has shown to decrease ethylene production, fruit softening, and loss of ascorbic acid during cold storage. According to Zhang et al. (2003), this is a consequence of lower ACC synthase and AA oxidase, key enzymes in the last step of ET synthesis. In addition, SA hindered ethylene production in cultured pear cells, mung bean hypocotyls, pear tissue disks, carrot cell suspension cultures, among others (Babalar et al., 2007).

Jasmonic acid is generally considered a systemic signal transducer for many physiological processes in the plant related to vegetative growth, cell cycle regulation, senescence, fruit ripening, and biosynthesis of many plant secondary metabolites, among others (Zhao et al., 2005; Sharma and Laxmi, 2016). According to Gill et al. (2013), Guo et al. (2013), Zhao et al. (2013), Dar et al. (2015), and Kazan (2015) JA induces the production of different chemicals involved in detoxification and redox balance in response to insect-driven wounding, pathogen attack, and environmental stresses, such as low temperature, salinity, heavy metal toxicity and latest, heat stress tolerance. Some of these are: proteinase inhibitors, antimicrobial secondary compounds, antioxidants, pathogenesis-related and cellular protection molecules, including proteins (Pauwels and Goossens, 2011; Sharma and Laxmi, 2016). The role of JA and ethylene signaling pathways regulating stress response and development processes have been shown to work in collaboration or in an antagonistic way (Zlotek et al., 2016). In contrast, Shinshi (2008) reported that depending on the type of stress, different defense responses can be induced through the sole activation of ethylene, or jasmonate, or both signaling pathways. In our study, JA increased along with ET, ABA, and SA when fruit developed moderate sun injury symptoms (**Figures 1A,C**, **2C,D**, **3A–D**). Interestingly, there is evidence showing a coordinated interaction between ABA and JA in response to water stress (dehydration) to protect the plant from it deleterious effects, specifically, JA would have a role regulating ABA biosynthesis and signaling network (Brossa et al., 2011; De Ollas and Dood, 2016). Changes in relative water content and tissue water potential, indication of dehydration stress, has been previously reported in sun injured fruit (Torres et al., 2013).

In contrast to SA, JA dramatically increased post-harvest (**Figures 5E,F**), probably signaling chilling injury protective mechanisms involving cryo-protectors, proteinase inhibitors, polyamines, ABA, and antioxidants, among others (Sharma and Laxmi, 2016). In fact, in agreement with our results, Du et al. (2013) reported an increase in JA levels upon exposure of rice to cold stress, as well as drought. In contrast, heat in this species reduced JA and increased IAA levels.

Recently, Hu et al. (2017) reported the induction of JA in response to cold stress in Arabidopsis, as well as the increase in freezing tolerance by exogenous applications of JA. The latest has also been found in other fruit and vegetable species using exogenous methyl JA, in which case an increase in secondary metabolites and antioxidant activity has been reported (Reyes-Díaz et al., 2016).

Our findings strongly suggest that there is a crosstalk between stress hormones (ET, JA, SA, ABA) modulating biochemical and physiological defense responses and (in some cases) acclimation (Ma and Cheng, 2003; Chen et al., 2008; Racsko and Schrader, 2012; Torres et al., 2013) during photooxidative stress (in the presence of elevated temperature) development on fleshy fruit such as apple. Nevertheless, once the fruit is harvested and subjected to another abiotic stress such as sub-optimal temperature during cold storage, the dramatic increase in JA in the tissue is responsible for activating another set of metabolic responses targeted to confer tolerance (with limited metabolic resources in sun-injured fruit) to this new abiotic stress.

## Conclusion

Photooxidative and heat stress on fruit tissue, leading to sun injury development, trigger a plethora of defense responses including antioxidant metabolites and enzymes as well as multiple secondary metabolites. Ethylene, abscisic acid, jasmonic acid and salicylic acid are differentially modulating stress response during this abiotic stress events that ultimately lead to losses in productivity and quality of the final product. Sunlight is causing IAA to decrease on sun exposed tissue while ET, ABA, JA, and SA increase dramatically on tissue that develops sun injury symptoms signaling photooxidative, heat and dehydration stress-defense mechanisms all together. A clear crosstalk between all these phytohormones are modulating the biochemical, physiological and structural changes in fruit tissue exposed to direct sunlight, which involve, initially, up-regulation of antioxidant metabolites and enzymes, secondary metabolites, specifically flavonols, osmolytes (carbohydrates, aminoacids, etc.). Despite all this metabolic machinery engaged through a complex hormonal regulation to protect the tissue from excess light and heat, oxidative stress depletes key antioxidants such as ascorbate and glutathione, leaving the tissue unable to acclimate to those harsh environmental conditions in the field, or later defense himself to chilling stress during cold storage.

## Author Contributions

CT: experimental and field design, discussion of results. GS: conducted field experiments and statistical analysis. BK: manuscript writing and discussion of results.

## Conflict of Interest Statement

The authors declare that the research was conducted in the absence of any commercial or financial relationships that could be construed as a potential conflict of interest.
